# Going Up Stairs

**Published:** 1879-09

**Authors:** 


HALL'S

### JOURNAL OF HEALTH.

### GOING UP STAIRS.

A MEMBER of a School Board writes that a building is about being erected for educational purposes, and wants to know the effect on pupils and teachers of climbing long flights of stairs.

An old man instinctively goes round a log instead of stepping upon it; each step up is equal to lifting a considerable portion of the body, and the old have no extra strength to spare, nor have the young and frail. The most robust are conscious of an effort in going up stairs, and we all "puff and blow" when we get to the top.

In New York City the young and vigorous are appointed to deliver letters in the lower part of the town, where they have to be taken up two or three or more stories of buildings containing business offices; they all complain of the exhausting effects; every now and then the carrier is missed; on inquiring the answer is, "dead." Others have had to give up at the end of three or four years and take positions for deliveries to private dwellings. If strong young men are thus worn out, so much more will children and female teachers. If they must go up stairs, each step should not be over six inches high, and should be at least twelve inches broad, so as to give support to the whole foot at each tread. There can be no argument against facts. In some cases weak lungs may be strengthened by going up stairs rapidly, with the mouth closed, so as to compel a deep inspiration when the top of the stairs is reached, and thus compel the air to the very bottom of the lungs, distending them fully ; but this, at the same time, endangers bleeding by the extra strain, if there is a tendency to hemorrhage or heart disease. The safest and best plan for all is to go up stairs slowly and deliberately, bearing in mind that, in all stages of life, equality of bodily motion and mental activity are most promotive of health. No person, after forty, should go on a run unless there is a seeming necessity for it. Running to catch a vehicle or a departing ferry boat is not only not dignified but is dangerous—always dangerous to young and old—and the more so as one is advanced in years, for, like going up stairs, it imposes an extra strain on the heart and lungs.

